# Study of alginate-encapsulated phycoerythrin in promoting the biological activity of synbiotic ice cream with *Lactobacillus casei*

**DOI:** 10.1038/s41598-024-66365-7

**Published:** 2024-07-05

**Authors:** Maryam Chamari, Seyed Amir Ali Anvar, Rezvan Pourahmad, Bahareh Nowruzi, Shima yousefi

**Affiliations:** 1 Department of Food Hygiene, Science and Research Branch, Islamic Azad University, Tehran, Iran; 2 Department of Food Science and Technology, Varamin–Pishva Branch, Islamic Azad University, Varamin, Iran; 3 Department of Biotechnology, Faculty of Converging Sciences and Technologies, Science and Research Branch, Islamic Azad University, Tehran, Iran; 4 Department of food Science and technology, Science and Research Branch, Islamic Azad University, Tehran, Iran

**Keywords:** Phycoerythrin, *Nostoc* sp., Antioxidant activity, *Lactobacillus casei*, Ice cream, Microbial activity, Probiotic ice cream, Biological techniques, Biotechnology, Microbiology, Health care

## Abstract

This study examines the effect of phycoerythrin (PE) from a cyanobacterial *Nostoc* strain encapsulated with alginate as a potential prebiotic to produce synbiotic ice cream products with *Lactobacillus casei*. It was found that the addition of the encapsulated PE affected, mostly favourably, the physicochemical properties, antioxidant activity, probiotic survival, volatile compound contents, and sensory acceptability of the synbiotic ice cream samples before and after aging at the freezing periods of one day to eight weeks. Thus, it confirms the prebiotic potential of PE for synbiotic ice creams with *L. casei*.

## Introduction

Phycoerythrin (PE) is a red pigment that is sensitive to light and belongs to the phycobiliprotein family and is mostly found in cyanobacterial species. Moreover, Phycoerythrin has a high degree of sensitivity towards fluctuations in pH, salt content, temperature deviations, desiccation, and light-induced stress^1,2^. On an equivalent molar basis, PE absorbs lighter than allophycocyanin (APC) and phycocyanin (PC) because it contains additional phycobilin. Only phycoerythrobilin (PEB) chromophores are commonly carried by PEs from freshwater and soil-dwelling cyanobacteria, and these PEs have absorbance spectra with maximum values of approximately 565 nm^3^. PEs generate fluorescence between 575 and 580 nm and substantially absorb light between 480 and 570 nm. PEs are divided into three primary kinds depending on phycobilin types and their spectral properties: (1) B-phycoerythrin (B-PE) (~ max ~ 540–560 nm, shoulder ~ 495 nm), (2) R-phycoerythrin (R-PE) (~ max ~ 565, 545 and 495 nm), and (3) C-phycoerythrin (C-PE) (~ max ~ 563, 543 and ~ 492 nm). Due to its flavour features, cooling impact, and beneficial nutritional characteristics, ice cream has emerged as one of the most extensively eaten dairy products across all age groups on a global scale^4^. The quality of the final product is often determined by the combination of milk, emulsifiers, stabilizers, sweeteners, and flavourings in the ice cream sample. The manufacturing process also impacts on the final quality of ice cream, where aging is essential to achieving the ideal flavour of ice cream^5^. The inclusion of fruits, spices, and ingredients high in protein, probiotics, and prebiotics may result in an ice cream that is more nutritional and healthier as a functional food. The positive functions of milk proteins, lipids, lactose, and other ingredients further contribute to the functionality of ice cream as a probiotic carrier food.

Probiotics are living bacteria that benefit consumers when taken in sufficient quantities^5^. Microbes must exhibit tolerance to gastrointestinal tract conditions, the capacity to auto-aggregate, behave normally, sustain flora, and be non-pathogenic. Probiotic products must comprise at least 10^7^ colony-forming unit CFU mL^-1^ of the probiotic strain^6^. Ice cream may be a perfect carrier for probiotic microbes because it combines milk proteins, lipids, lactose, and various components. The production of probiotic ice cream using different formulations has been the subject of a limited number of studies^7–11^. The most popular probiotic bacteria in probiotic dairy products are *lactobacilli*, and to a lesser extent, *Bifidobacteria* microbial strains^6^.

Prebiotics consist of indigestible carbohydrates, mostly oligosaccharides, which promote the development or activity of certain probiotic bacteria in the colon, therefore benefiting the host organism. Microalgae are well recognized as a significant reservoir of sulfated polysaccharides, with the specific composition of these polysaccharides exhibiting variation across different taxonomic groups. The primary role of these polysaccharides with relatively high molecular weight is their abundance of hydroxyl (OH) groups, which renders them hydrophilic. These entities have been seen to form intra-chain hydrogen bond networks, resulting in increased stiffness, rigidity, and suitability as thickening agents. The consistent arrangement of their structures also facilitates their engagement with external ions and inter-chain hydrogen bonding, such as the process of gelation. The food and pharmaceutical sectors often use various types of carbohydrates, such as ulvans, carrageenan, and alginates, derived from macroalgae as functional additives^12^. Prebiotic substances may be utilized in product compositions to stimulate the development and viability of probiotics. The research on ice cream formulations has mostly included dietary fibers and oligosaccharides such as inulin as prebiotic ingredients^6,13,14^. Further, algal biomass and its derivatives, such as PE, may be used to make synbiotic ice cream products because of the abundance of colours and other substances that provide stabilizing functions as a sustainable alternative to artificial food dyes^4,15,16^.

Alginate, a biodegradable polymer, frequently serves as an encapsulating material due to its ability to form gel beads in a solution at ambient temperature without the need for organic solvents. Since it is non-toxic and often utilized as a suitable cross-formation with polymer alginate microspheres. The encapsulation of the PE and bacteria by alginate is an innovative method to protect their integrity^5,11^ in harsh processing conditions^9^. For example, De Oliveira et al.^17^ encapsulated a *Spirulina* sp. cyanobacterial strain with maltodextrin and soy lecithin, compared to alginates in chocolate milk samples, while Tiepo et al.^18^. encapsulated *S. platensis* with maltodextrin and Arabic gum in ice cream^18^. Ganesan and Shanmugam (2020) pioneered the use of algal PE in ice cream production. Their study employed PE extracted from the macroalgal strain, *Kappaphycus alvarezii*, biomass^19^. They also encapsulated it with a macroalgal derivative, kappa-carrageenan, as well as guar gum to protect the integrity of the PE during the processing. The results showed that ice cream enriched with the encapsulated PE had better rheology, and the intensity of the pink colour increased during 90 days of storage. Further, this sample had better antioxidant activity, and the viability of this PE was confirmed. Further, Ganesan et al.^20^ reviewed the extraction, encapsulation, and application of the PE from macroalgae in foods^20^. The focus was on the macroalgae-based PEs and their extraction and encapsulation since it is necessary to protect the integrity of the PE in the harsh ice cream processing conditions. In addition, Garcia et al.^2^ used a B-PE extract from the microalgal *Porphyridium cruentum* strain as a natural food colorant in milk-based products^2^. They focused on the colour stability of the PE in the milk products.

The cyanobacterium *Nostoc* sp. is a photoautotrophic bacteria that is found in abundance in nature. It has been used as a dietary supplement by humans for millennia due to its well-known nutritional benefits. The food and medicinal sectors have used several algae species, including *Spirulina platensis*, *Chlorella* spp, *Tetraselmis* sp. and *Dunaliella salina*. However, there is a scarcity of studies evaluating *Nostoc* sp. as a prebiotic bacterium.

To our knowledge, this is the first-ever study to examine the potential of PE from a cyanobacterial *Nostoc* strain encapsulated with alginate to protect its integrity in harsh processing environments in ice cream with the *L. casei* strain.

Therefore, bacterial strains and alginate-encapsulated PEs were introduced either alone or in a mixed culture to the control sample before or after aging to create probiotic and synbiotic ice cream in a range of five ice cream samples: control sample (C), probiotic sample before aging (LBA), synbiotic sample before aging (PLBA), probiotic sample after aging (LAA), and synbiotic sample after aging (PLAA). Moreover, the physicochemical properties, antioxidant activity, probiotic survival, volatile compound (VC) contents, and sensory acceptability of the probiotic and synbiotic ice cream samples before and after aging at the freezing periods of one day to eight weeks were studied in detail.

This study investigated the development of synbiotic ice cream. We incorporated *Lactobacillus casei* and alginate-encapsulated phycoerythrin from *Nostoc* sp. ASN_M into the ice cream mix, evaluating the impact on probiotic viability during storage (eight weeks). We assessed the effects of these additions on various technological properties, including physicochemical characteristics, overrun, color, melting rate, rheology, volatile compound formation, antioxidant activity, and sensory attributes. Our findings are presented and discussed in relation to existing literature. Additionally, we propose directions for future research in this field.

## Materials and methods

### Cyanobacterial biomass isolation and growth

Cyanobacterial *Nostoc* sp. ASN_M strain was obtained from paddy fields in the Golestan region of Iran (36.54.41 N, 54.47.25 W). Soil samples were distributed in sterile Petri dishes comprising liquid BG-11 medium. To obtain a cyanobacterial monoculture, the medium was made nitrogen-free with a pH of 7.1 and incubated for two weeks at 28 ± 1 °C under constant artificial illumination of 1500–2000 lx. Selected colonies were moved to a new solid medium after 14 days of development. For bacterial-free cultures, colonies were examined for bacterial contamination on caseinate-glucose agar and dextrose-peptone broth. On various agar slants, the chosen bacterial-free colony was kept alive. After 20 days, the isolate was given a last wash in sterile deionizer water and put into 1 L of newly made liquid BG110 medium.

### Chemicals and alginates

Indicators of protein molecular weight and other analytical components were purchased from Hi-Media, Merck, and Sigma, respectively. Double-distilled water was used in the production of all reagents and buffers. The alginate and the CaCl_2_ were obtained from a local grocery store and were safe for human consumption.

### Extraction and purification of phycoerythrin

To extract phycoerythrin (PE), approximately 500 mL of a homogenized, 14-day-old log-phase culture was centrifuged at 4000 rpm after resuspending the pellet in 100 mL of 20 mM acetate buffer (pH 5.1). The cell suspension was subjected to repeated cycles of freezing (− 20 °C) and thawing at room temperature over four days, until the supernatant turned dark purple. Following centrifugation at 5,000 rpm for 10 min, the resulting crude extract was further purified according to the method described by Afreen and Fatma^21^. The crude extract was gradually added to a constant stream of solid ammonium sulphate until it reached 65% saturation. The solution was centrifuged at 4500×*g* for 10 min after standing for 12 h in a cool environment. The pellets were dialyzed overnight after being re-suspended in a tiny amount of 50 mM acetic acid-sodium acetate buffer (pH 7.1). A 0.45 μm filter removed the extract from the dialysis membrane^22^. The Specord 200 spectrophotometer (Analytik Jena, Germany) was used to scan the sample in the 300–750 nm wavelength range and to acquire the absorption spectra.

Absorbance at 565, 620, and 650 nm was used to determine the concentrations of PE, PC, and APC in various extracts and PBPs containing solutions following De Oliveira et al.^17^. The purity ratio (A562/A280) was used to evaluate PE purity at each step, and the absorbance at 562 nm was used to quantify PE concentration.

$$\text{PC }(\upmu \text{g mL}^{-1}) =\frac{(\text{OD } \, 620\text{nm}-0.7\text{OD }650\, \text{nm})}{7.38}$$

$$\text{APC }(\upmu \text{g mL}^{-1}) =\frac{(\text{OD }650\, \text{nm}-0.19\text{OD}620\, \text{nm})}{5.65}$$

$$\text{PE }(\upmu \text{g mL}^{-1}) =\frac{(\text{OD }565\, \text{nm}-2.8\left[\text{R}-\text{PC}\right]-1.34\left[\text{APC}\right])}{12.7}$$

### Toxicity assays of PE

Wild-type *C. elegans* var. Bristol-N2 was gifted from the Pasteur Institute of Tehran, Iran. Nematode growth agar was used to culture *C. elegans*. Eggs were deposited on agar plates containing nematode growth agar (NGA, Biomol, USA) for 48 h, and young larvae were harvested and suspended in M9 worm buffer. The toxicity test of the PE was evaluated at concentrations of 0, 0.125, 0.2, 0.5, and 1 mg/ml. The nematode without PE was used as a control. Quadruplicate tests were carried out for 24 h at 18 °C^23^.

### Encapsulation of phycoerythrin by alginate

The alginate solution was made at a 3% (w/v) concentration. Alginate and PE were combined in a 1:1 (w/w) ratio, and the mixture was then thoroughly combined by implementing a magnetic stirrer. The homogenized suspension was loaded into the syringe, injected into a 2.5% (w/v) CaCl_2_ solution with a 23G needle, and agitated with a magnetic stirrer. The beads were collected and rinsed with distilled water to eliminate Cl ions. To compute the encapsulation efficacy, CaCl_2_ and distilled water were gathered, and the beads were refrigerated. Before and after the aging stages, 0.4 g of encapsulated PE was added to 150 g of pasteurized ice cream mixture at 37 °C. The encapsulated PE beads were not dehydrated before being added to the ice cream mix.

### Probiotic strain cultivation and preparation

*L. casei* microbial strain was cultivated overnight at 30 °C to produce 10^8^ CFU mL^−1^ of bacterial cells in MRS broth (Merck, Germany). The inoculated broth was centrifuged at a speed of 4500×*g* for 30 min, at 4 °C to separate the bacteria from the MRS broth. The broth was completely removed by centrifuging the bacterial pellet with peptone water (0.1%, w/v) at 4500×*g* for 10 min at 4 °C. This technique was carried out three times. Peptone water and the bacterial pellet devoid of broth were combined. Before and after aging, a mixture comprising 10^8^ CFU mL^−1^ of bacterial cells was added (1%, v/v) to a mixture of pasteurized ice cream at 37 °C.

### Production of ice cream

**Figure 1:**
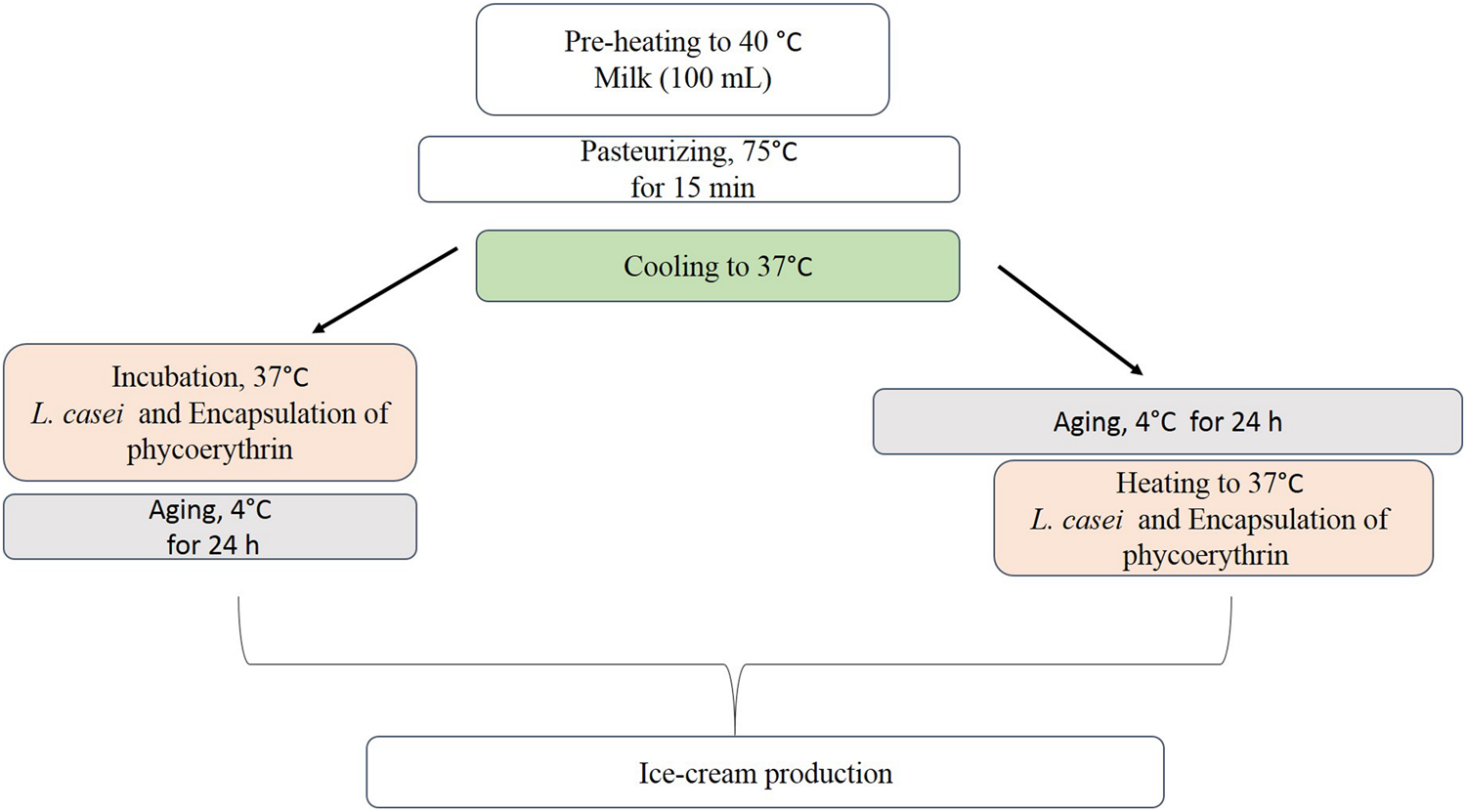
Production flow chart of probiotic ice cream samples. Five different ice cream samples were produced in total: *C* control sample, *LBA* probiotic sample before aging, *LPBA* synbiotic sample before aging, *LAA* probiotic sample after aging, *LPAA* synbiotic sample after aging.

**Figure 2:**
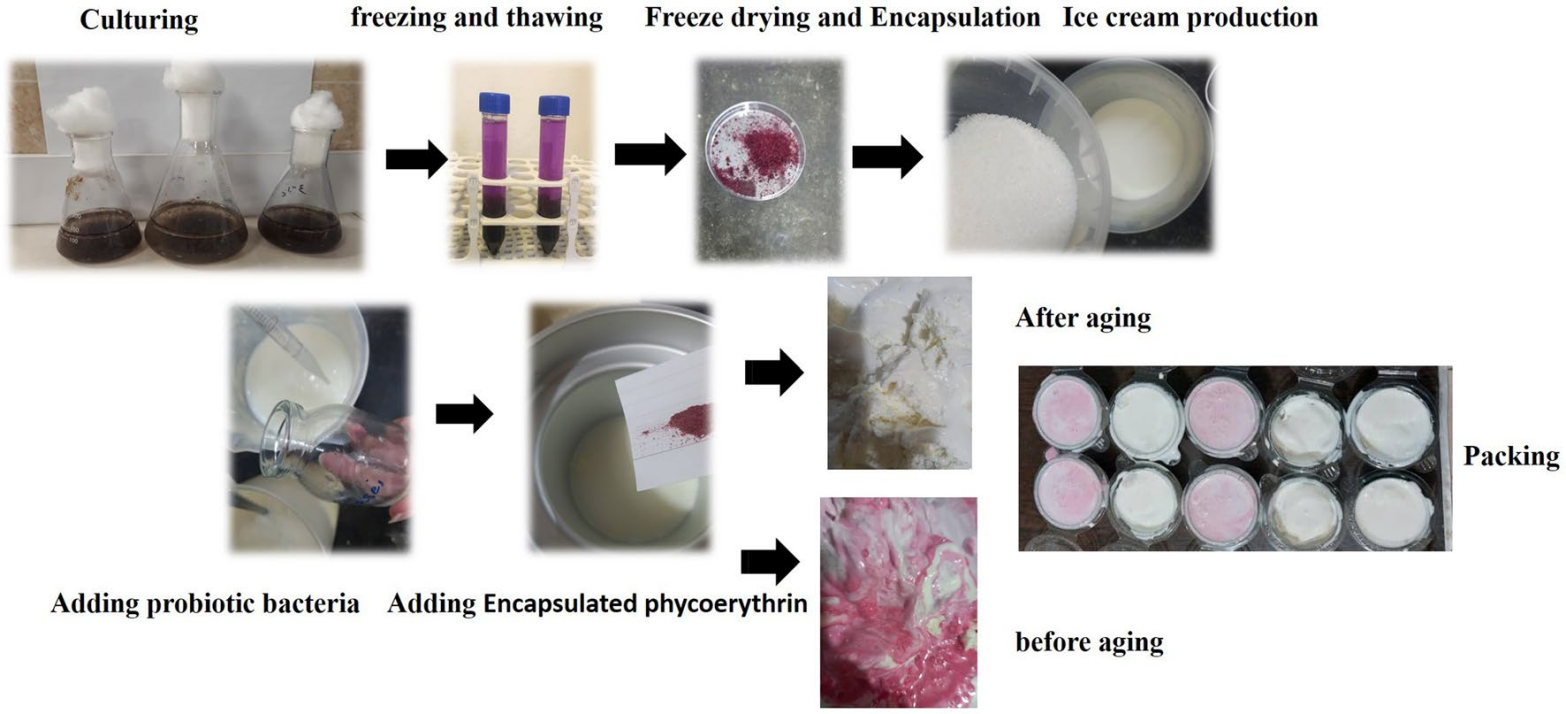
Phycoerythrin production process and adding the probiotic *L. casei* strain before or after aging steps.

Figures 1 and 2 shows the production process. First, the raw milk was pre-heated to 40 °C and then pasteurizing in 75 °C for 15 min. Then, after the cooling process to about 37 °C, the incubation and heating of *L. casei* and PE-encapsulation was performed at 4 °C for 24 h. The ice cream ingredients, such as sugar, sahlep, and emulsifier, were annexed in accordance with the recipe given in the scheme, and sahlep was utilized as a stabilizer. The ice cream products were produced in 500 mL of ice cream mix using a Delonghi II Gelataio ICK5000 ice cream machine (China), and the method was adapted previously described by Durmaz et al.^4^. The ice cream production facility conducted probiotic and synbiotic ice cream trials, adding probiotic cultures to the ice cream mixture and agitating it for 30 min at room temperature before using the ice cream machine. Furthermore, samples of ice cream made with prebiotics were incubated for 24 h at 4 °C.

### Determination of physicochemical properties

On the first day, ice cream samples were subjected to physicochemical analyses, including titratable acidity, pH, protein, fat, ash, and overrun, employing AOAC techniques 947.05, 981.12, 930.33, 952.06, 930.30, and 968.14, respectively^24^.

The melting properties of ice cream products were characterized on day one using a TA Instrument Q100 (New Castle, USA) and the DSC technique employing ice cream samples. In hermetically sealed aluminium containers, the thermal performance of 10–15 mg of ice cream samples was assessed between 30 and 20 °C^25^. The total sugar content was calculated by the Fehling technique following Iranian Standard 2450.

Each sample of probiotic and synbiotic ice cream was assessed for its viscosity by applying a viscometer (Brookfield RH, USA) after it had settled at 25 ± °C for 20 h. The dry matter percentage of ice cream samples was obtained utilizing the AOAC techniques )941.08( and the moisture content was measured using the AOAC techniques (at one day) and after 2, 4, 6, and 8 weeks^24^.

### Assessment of DPPH radical scavenging activity

The method by Sagdic et al.^26^ was adapted to produce the extracted materials. In this study, 5 g of each sample was mixed with 25 mL of a 75% methanol solution and then stored at 25 °C for 24 h. A pure extract for spectrophotometric analysis was obtained by filtering the mixture through a membrane filter with a diameter of 0.45 μm. Then, extracts were kept at 4 °C while their total phenolic contents (TPC) and antioxidant activities were tested. The Folin–Ciocalteu technique was used with a few modifications to determine the amount of TPC in sample extracts^27^. Ice cream extract in 0.1 mL was combined with Folin-Ciocalteu reagent (Merck, Germany), 20% sodium carbonate (w/v), 1.5 mL, and 7.9 mL of distilled water. Two hours were spent maintaining the mélange in the dark at 25 °C. The absorbance at 750 nm was checked after incubation. Using the gallic acid (anhydrous form, Merck, Germany) curve, the findings were expressed in milligrams (gallic acid equivalents, GAE) per 100 g of ice cream samples.

All samples were subject to a TPC examination. The 1,1-diphenyl-2-picrylhydrazyl radical (DPPH) experiment was carried out using a modified version of the technique to assess the antioxidant potential of ice cream extracts^28^. The free radical form of DPPH (90% purity) was obtained from Sigma-Aldrich (USA). We employed a DPPH radical scavenging assay to determine the extract concentration required for 50% inhibition (EC50 value). This assay involved plotting the percentage of DPPH inhibition against various extract concentrations. Changes in antioxidant activity were evaluated after one day, two weeks, four weeks, six weeks, and eight weeks of frozen storage at – 20 °C.

### Assessment of the probiotic survival in ice creams

After one day, two weeks, four weeks, six weeks, and eight weeks of freezing at − 20 °C viable bacterial counts were determined for five distinct ice cream samples of C, LBA, LPBA, LAA, and LPAA. *L. casei* was enumerated using MRS agar (Merck, Germany) supplemented with 0.05% vancomycin (Sigma-Aldrich, United States). At 30 °C plates were incubated for two days in anaerobic conditions^6^. To compute colony counts, log CFU g^−1^ was converted.

### Consumer sensory evaluation

Following Lawless and Heymann^29^, all ice cream samples were subjected to sensory assessment after a week of storage. Samples of ice cream were examined by employing a nine-point hedonic scale, with values varying from 1 (extremely dislike) to 5 (extremely like). Eleven panellists with limited to no prior knowledge had attended training sessions at Selcuk University's Food Engineering Department, where specific feature explanations were presented and sensory acceptability testing was performed.

### Ice cream product volatile compound (VC) analysis

The volatile compounds (VCs) present in the control (C) and synbiotic LPBA ice cream samples were profiled after one week of freezing using gas chromatography-mass spectrometry (GC–MS). This technique separates and identifies the individual VCs within a sample. An Agilent 7000 Quadrupole GC–MS system with electron impact ionization was employed for analysis. By comparing the obtained mass spectra to databases like the NIST library (containing over 62,000 patterns) and the Fiehn Mass Spectra Libraries, the identities, molecular weights, and structures of the VCs were determined.

### Statistical analysis of data

Using Excel and SPSS software (version 24), statistical analysis of the data gathered from every experiment was conducted. All data were generated by experimenting three times. Significant discrepancies between the components were discovered using a one-way analysis of variance with a 95% confidence interval (< 0.05). Subsequently, the Tukey test was used to compare the means, and then Excel was used to present the results of our analyses graphically.

### Statement

All procedures were carried out following applicable rules and guidelines. The authors did not conduct human experiments or use human tissue samples.

## Results

### Extraction, purification, and characterization of phycoerythrin

**Figure 3:**
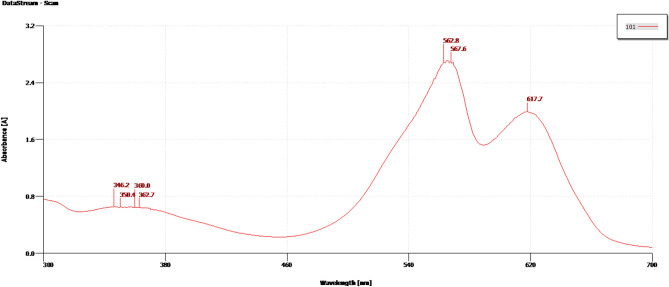
Spectra of PE extracted and purified from *Nostoc* sp. ASN_M, spectra show absorption maxima at 562 nm.

The PE content and purity of the ice cream samples were examined at each stage of purification, as indicated in Table 1. The PE purity ratio rose from 0.797 to 3.20, while the PE content increased from 0.108 to 0.193 μg/mL during the purification processes. The reddish-pink-coloured protein precipitated in the buffer displayed absorption maxima at 562 nm with a shoulder peak at 617, a characteristic spectrum for PEB containing PE (Fig. 3).

**Table 1:** Stepwise purification of phycoerythrin from *Nostoc* sp. ASN_M biomass.

Steps	Peak (nm)	PE (μg/mL)	PR
*Nostoc* sp. ASN_M			
Crude extract	566.2–616.9	0.108	0.797
Ammonium sulphate precipitation	565.5–617.4	0.1518	1.559
Dialysis	567.6–617.7	0.193	3.20

*PR* Purity ratio of PE (OD562/OD280).

### Toxicity of PE against *C. elegans*

*C. elegans* survival rates were not affected by PE, with 100% to 96.1% survival at all concentrations used (Table 2).

**Table 2:** Toxicity of phycoerythrin against the nematode *Caenorhabditis elegans*.

mg/ml	Phycoerythrin
	% survival
0	100 ± 0.71^a^
0.125	96.5 ± 0.21^a^
0.2	98.3 ± 0.11^a^
0.5	98.3 ± 0.07^a^
1	92.1 ± 0.09^a^

Mean ± SE, n = 3. The same letter indicates that data are not significantly different from each other (P < 0.05).

### Physicochemical qualities of ice cream samples

Table 3 displays the physicochemical characteristics of five ice cream samples. This table shows that protein content (%), fat content (%), ash (%), overrun (%), total sugar (%), melting (g/min), and viscosity (cP) did not differ significantly between samples (P > 0.05).

**Table 3:** Physicochemical features of ice cream samples before and after aging.

No.	Sample	Protein content (%)	Fat content (%)	pH	Ash %	Overrun (%)	Total sugar (%)	Melting rate (g/min)	Titratable acidity (%)	Viscosity (cP)
1	C	7.15 ± 0.15a	2.80 ± 0.06a	6.66 ± 0.00a	0.99 ± 0.01a	22.67 ± 0.33ab	14.64 ± 0.10a	2.44 ± 0.01a	0.15 ± 0.00a	243.67 ± 3.53ab
2	LBA	7.15 ± 0.15a	2.73 ± 0.03a	6.44 ± 0.01b	1.00 ± 0.01a	21.00 ± 0.58ab	14.46 ± 0.10a	2.44 ± 0.00a	0.16 ± 0.00a	246.00 ± 1.15ab
3	LPBA	7.29 ± 0.15a	2.73 ± 0.03a	6.32 ± 0.01c	1.01 ± 0.01a	20.67 ± 0.33a	14.40 ± 0.06a	2.42 ± 0.01a	0.17 ± 0.00b	252.33 ± 1.76b
4	LAA	7.15 ± 0.15a	2.77 ± 0.03a	6.62 ± 0.01d	1.00 ± 0.00a	23.00 ± 0.58b	14.64 ± 0.10a	2.43 ± 0.00a	0.16 ± 0.00a	242.67 ± 0.88a
5	LPAA	7.29 ± 0.15a	2.77 ± 0.07a	6.59 ± 0.01e	1.01 ± 0.01a	21.67 ± 0.33ab	14.58 ± 0.06a	2.42 ± 0.01a	0.16 ± 0.00a	246.33 ± 1.20ab

*C* Control sample, *LBA* Probiotic sample before aging, *LPBA* Synbiotic sample before aging, *LAA* Probiotic sample after aging, *LPAA* Synbiotic sample after aging. Small letters show the significant differences of mean in the columns (P < 0.05).

However, the titratable acidity and pH values of the C sample were substantially distinct (P < 0.05) from those of the other samples because of the fermentation method.

The C and synbiotic LPBA samples had the highest and lowest pH values, respectively, while the pH values of the other samples varied from 6.66 ± 0.00 to 6.59 ± 0.01 (Table 3).

Further, the overall solid content increased slightly in the probiotic and synbiotic ice cream samples for all the freezing periods, from one day to eight weeks (Table 4). However, the moisture contents decreased in the synbiotic ice cream samples throughout all the freezing periods (Table 5).

**Table 4:** Total solid content of ice cream samples before and after aging.

	C	LBA	LPBA	LAA	LPAA
Day 1	33.54 ± 0.93 (a)A	33.64 ± 0.05 (b)A	33.97 ± 0.05 (bc)A	33.62 ± 0.07 (cd)A	34.01 ± 0.03 (d)A
Two weeks	33.81 ± 0.04 (a)B	33.82 ± 0.01 (a, b)AB	34.22 ± 0.08 (b, c)AB	33.84 ± 0.02 (c, d)AB	34.26 ± 0.11 (d)AB
Four weeks	34.002 ± 0.03 (a)BC	33.95 ± 0.07 (a, b)BC	34.39 ± 0.06 (b, c)BC	34.02 ± 0.06 (c, d)BC	34.33 ± 0.08 (d)B
Six weeks	34.18 ± 0.06 (a)CD	34.15 ± 0.11 (a, b)CD	34.64 ± 0.01 (b, c)CD	34.17 ± 0.04 (c, d)CD	34.57 ± 0.02 (d)BC
Eight weeks	34.30 ± 0.052 (a)D	34.30 ± 0.034 (ab)D	34.82 ± 0.04 (b)D	34.26 ± 0.02 (bc)D	34.83 ± 0.02 (c)C

*C* Control sample, *LBA* Probiotic sample before aging, *LPBA* Synbiotic sample before aging, *LAA* Probiotic sample after aging, *LPAA* Synbiotic sample after aging. Small and capital letters show the significant differences of mean in the rows and columns (P < 0.05).

**Table 5:** Moisture contents of ice cream samples before and after aging.

	C	LBA	LPBA	LAA	LPAA
Day 1	66.45 ± 0.093 (a)A	66.36 ± 0.051 (a)A	66.02 ± 0.055 (b)A	66.38 ± 0.07 (a)A	65.98 ± 0.031 (b)A
Two weeks	66.18 ± 0.04 (a)B	66.17 ± 0.015 (a)AB	65.77 ± 0.083 (b)AB	66.15 ± 0.02 (a)AB	65.73 ± 0.11 (b)AB
Four weeks	66.00 ± 0.034 (a)BC	66.04 ± 0.07 (a)BC	65.61 ± 0.06 (b)BC	65.98 ± 0.06 (a)BC	65.67 ± 0.08 (b)B
Six weeks	65.82 ± 0.06 (a)CD	65.85 ± 0.11 (a)CD	65.36 ± 0.016 (b)CD	65.82 ± 0.04 (a)CD	65.43 ± 0.02 (b)BC
Eight weeks	65.70 ± 0052 (a)D	65.70 ± 0.34 (a)D	65.18 ± 0.041 (b)D	65.73 ± 0.02 (a)D	65.17 ± 0.02 (b)C

*C* Control sample, *LBA* Probiotic sample before aging, *LPBA* Synbiotic sample before aging, *LAA* Probiotic sample after aging, *LPAA* Synbiotic sample after aging. Small and capital letters show the significant differences of mean in the rows and columns (P < 0.05).

### Evaluation of changes in antioxidant activity

Table 5 illustrates the EC_50_ values for the DPPH radical-scavenging abilities of the ice cream samples. It was determined that symbiotic LPBA samples had the highest radical scavenging activity and the lowest EC_50_ value across the freezing periods, followed by the probiotic ice cream samples and the synbiotic LPAA sample. On the contrary, at week eight, the C sample had the lowest radical scavenging activity and the maximum EC_50_ value (P < 0.05; Table 6).

**Table 6:** EC_50_ values of the ice cream samples before and after aging.

	C	LBA	LPBA	LAA	LPAA
Day1	56.79 ± 0.54 (a)A	44.05 ± 0.22 (b)A	34.32 ± 0.2 (c)A	45.48 ± 0.72 (d)A	41.59 ± 0.14 (e)A
Two weeks	67.08 ± 0.47 (a)B	52.90 ± 0.29 (b)B	37.29 ± 0.14 (c)B	55.36 ± 0.24 (d)B	45.91 ± 0.22 (e)B
Four weeks	71.69 ± 0.35 (a)C	55.40 ± 0.38 (b)C	40.13 ± 0.29 (c)C	58.95 ± 0.24 (d)C	49.45 ± 0.37 (e)C
Six weeks	83.07 ± 0.86 (a)D	61.61 ± 0.42 (b)D	44.13 ± 0.26 (c)D	63.58 ± 0.33 (d)D	55.56 ± 0.25 (e)D
Eight weeks	90.62 ± 1.05 (a)E	63.61 ± 0.35 (b)E	47.51 ± 0.28 (c)E	66.33 ± 0.46 (d)E	60.03 ± 0.37 (e)E

*C* Control sample, *LBA* Probiotic sample before aging, *LPBA* Synbiotic sample before aging, *LAA* Probiotic sample after aging, *LPAA* Synbiotic sample after aging. Small and capital letters show the significant differences of mean in the rows and columns (P < 0.05).

### Evaluation of probiotic survival and prebiotic potential

Table 7 lists the bacterial counts found in five samples of ice cream. After eight weeks of freezing, the bacterial quantities in all samples decreased compared to the other freezing periods (P < 0.05). On the other hand, the probiotic and synbiotic samples had significantly higher bacterial counts for all the freezing periods, while the synbiotic LPBA samples had the highest bacterial counts for all the freezing periods. In other words, the synbiotic samples (LPBA and LPAA) had higher bacterial counts compared to the probiotic samples (LBA and LAA), respectively. Further, the after-aging LAA and LPAA samples had the lowest bacterial counts compared to the before-aging LBA and LPBA samples, respectively, for all the freezing periods. In other words, aging resulted in the lowest bacterial counts.

**Table 7:** *L. casei* counts of the ice cream samples before and after aging.

	C	LBA	LPBA	LAA	LPAA
Day1	3.66 ± 0.03 (a)A	9.87 ± 0.03 (c)A	10.15 ± 0.02 (d)A	8.53 ± 0.03 (b)A	8.53 ± 0.02 (b)A
Two weeks	3.72 ± 0.03 (a)B	10 ± 0.01 (d)B	10.26 ± 0.01 (e)B	8.65 ± 0.02 (c)B	8.72 ± 0.03 (b)B
Four weeks	3.76 ± 0.01 (a)C	10.20 ± 0.06 (d)C	10.37 ± 0.02 (e)C	8.79 ± 0.03 (c)C	8.85 ± 0.01 (b)C
Six weeks	3.56 ± 0.05 (a)D	9.77 ± 0.02 (d)D	10.21 ± 0.03 (e)D	8.58 ± 0.04 (b)D	8.68 ± 0.08 (c)D
Eight weeks	3.38 ± 0.03 (a)E	8.71 ± 0.07 (d)E	9.31 ± 0.05 (e)E	7.47 ± 06 (b)E	7.95 ± 0.06 (c)E

*C* Control sample, *LBA* Probiotic sample before aging, *LPBA* Synbiotic sample before aging, *LAA* Probiotic sample after aging, *LPAA* Synbiotic sample after aging. Small and capital letters show the significant differences of mean in the rows and columns (P < 0.05).

### Sensory acceptability of ice cream samples

**Figure 4:**
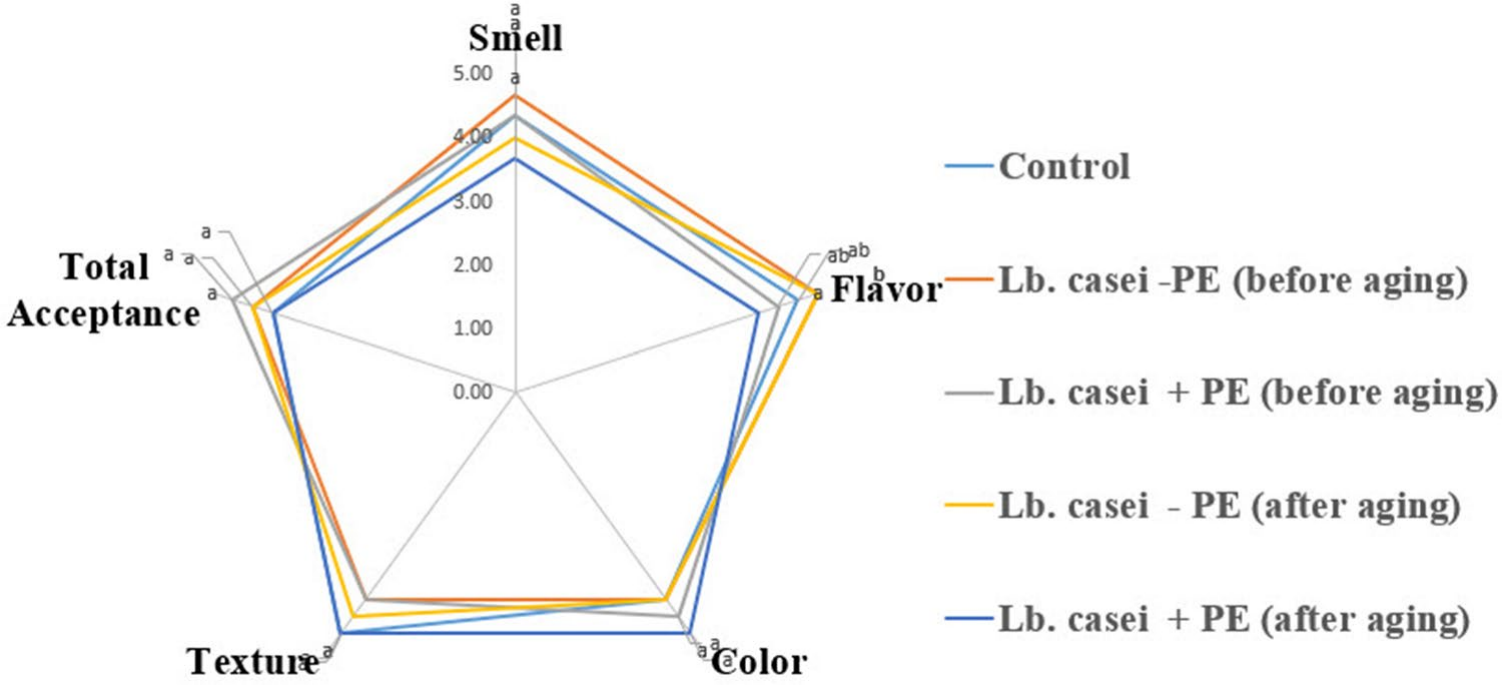
Sensory scores of ice cream products produced with PE and *L. casei* according to the sensory acceptability test before and after aging.

The sensory qualities of ice cream products in terms of flavour, colour, texture, and general acceptance are illustrated in detail in Fig. 4. The appearance, crystalline structure, and dissolving characteristics of the probiotic LBA and LAA samples were comparable to those of the C sample. However, the aroma, flavour, colour, texture, or total acceptance ratings of the ice cream samples varied from one another (P > 0.05). The probiotic LBA sample was the one with the best flavour and aroma. Nevertheless, compared to the C sample, the synbiotic LPBA and LPAA samples had poorer ratings for aroma, flavour, colour, texture, and overall acceptability attributes (P ≤ 0.05). However, the symbiotic LPBA sample had a higher general acceptance score than the C sample.

### Volatile compound (VC) profiles of synbiotic ice cream samples

The C sample comprised 25 VCs (aldehyde, ketone, ester, and alcohol), according to the results of the GC–MS characterization of the VCs in ice cream samples. In comparison, the synbiotic LPBA sample contained 27 VCs (aldehyde, ketone, ester, and alcohol). The most common VCs found in the C sample were acetone (23.55%), ethanol (15.47%), 3_methyl butanal (4.95%), 2_3_butanedione (5.69%), 3-methyl-1-butanol (5.24%), and 1-hexene (4.82%). The VCs found in the synbiotic LPBA sample, on the other hand, were mostly acetone (17.97%), ethanol (9.76%), acetaldehyde (7.48%), isobutyraldehyde (5.89%), and 3-methyl-1-butanol (4.81%). In general, the symbiotic LPBA sample had more or less VCs compared to the C sample, as shown in Table 8.

**Table 8:** Comparison the volatile compounds of the control and synbiotic LPBA samples.

Name of compounds	Peak IDs	RT (min)	Abundance %	RT (min)	Abundance %	Decrease or increase in comparison to control
	Nature of compounds	Control		LPBA		
1—Acetaldehyde	Aldehyde	4.35	3.315076	4.35	7.483499	Increase
2—Ethanol	Alcohol	5.74	15.47327	5.74	9.768229	Decrease
3—Acetone	Ketone	6.74	23.55803	6.74	17.97106	Decrease
4—Bis(Methylethio)methane	Alcohol	8.12	4.38388	8.12	3.239072	Decrease
5—Methyl acetate	Ester	8.56	1.523138	8.56	2.476296	Increase
6—Dimethyl sulfone	Sulfone	9.06	1.30112	9.06	2.301247	Increase
7—Isobutyraldehyde	Aldehyde	9.85	1.307492	9.85	5.890296	Increase
8—2_3_Butanedione	ketone	11.13	1.448706	11.13	1.059827	Decrease
9—1_Hexene	Alken	11.4	2.726569	11.4	2.461327	Decrease
10—2_Butanone	Ketone	11.45	5.891043	11.45	4.038442	Decrease
11—Ethyl acetate	Ester	12.6	3.760377	12.6	4.026322	Increase
12—Isobutanol	alkyl alcohol	13.1	1.059414	13.1	1.475306	Increase
13—3_Methyl butanal	Aldehyde	14.81	5.955264	14.81	8.909839	Increase
14—2_Methyl butanal	Aldehyde	15.25	2.1599	15.25	1.580239	Decrease
15—2_Pentanone	Ketone	16.1	1.454777	16.1	1.122042	Decrease
16—Pentanal	Alcohol	17.02	1.15374	17.02	1.511427	Increase
17—Ethyl propionate	Ester	17.65	3.021329	17.65	1.274021	Decrease
18—3-Methyl_1_butanol	Alcohol	18.77	5.247783	18.77	4.818658	Decrease
19—2_Hexanone	Ketone	19.05	1.061724	19.05	1.072938	Increase
20—1_Pentanol	Aldehyde	20.55	1.618284	20.55	1.728029	Increase
21—Toluene	Benzen	21.01	1.564111	21.01	3.159226	Increase
22—Hexanal	Aldehyde	22.2	2.714409	22.2	4.522019	Increase
23—2_Heptanone	Ketone	26.2	2.043201	26.2	1.320495	Decrease
24—Heptanal	Aldehyde	27.05	1.431417	27.05	1.777608	Increase
25—Alpha_pinene	Terpene	–	–	29.1	1.742399	–
26—6_Methyl_5_heptone_2_one	Ketone	–	–	30.04	1.825988	–
27—n_butanol	Ketone	–	–	15.05	1.444151	–

*LPBA* Synbiotic sample before aging.

## Discussion

Ice cream is a popular dairy product enjoyed by people of all ages around the globe. It is appreciated for its delicious taste, refreshing qualities, and nutritional benefits. The food and beverage industry is currently exploring natural dyes as alternatives to synthetic dyes due to concerns regarding their toxicity, carcinogenicity, and safety when added to food products at certain concentrations. The increasing demand for certain food products is driven by evolving consumer preferences. Nowadays, consumers are seeking out natural foods that undergo minimal processing and offer health benefits through bioactive properties, such as antioxidant effects. Nevertheless, the application of natural dyes in food is constrained by the characteristics of the food matrix in which the pigment is distributed, as well as its interactions with other components like proteins, polysaccharides, lipids, sugars, salts, and more^30,31^. The primary aim of this study is to develop an ice cream that is enriched with cyanobacterial pigment. The ice cream subjected to microbial, physiochemical, and antioxidant analysis. Additionally, the ice cream evaluated for its synbiotic properties with *Lactobacillus casei*.

The Extraction and purification results of our study showed that both PE content and purity of the ice cream samples increased to almost four times from crude extract to purified PE, illustrating the process's effectiveness in producing high-purity PE. In comparison to our study, in the previously article by Nowruzi et al.^22^, the purified C-phycoerythrin resulted in the purification of 65.04 µg mL^−1^ via acetate buffer (pH 5.1). In another study by the same author, the concentration and purity of extracted PE from *Nostoc* sp. were increased after each purification step by almost four times (from 1.5 to 6.22)^32^.

Adding algal biomass and their derivatives such as PE to ice cream changes its physicochemical properties such as ice cream mix, particularly pH and acidity as they raise the proportion of solids-non-fat in the combination. During storage, lactic acid is produced by lactic acid bacteria and causes the consumption of existing lactose and the production of hydrogen. The decrease in pH occurs due to the production of acid by lactic acid bacteria and the increase in acidity. During their numerous studies, researchers have stated that the activity of starter bacteria causes a significant drop in pH during the storage period. The pH scale is displayed as the negative logarithm of the molar concentration of hydrogen ions, and as the strength of the acid or hydrogen ions increases, the pH scale decreases and determines the concentration of hydrogen ions or ionized hydrogen in the sample. For example, in our study the LBA and LPBA samples had lower pH values compared to the C and LAA and LPAA samples. Our findings were consistent with the related studies, which indicated that probiotic ice cream samples had lower pH values (Alamprese et al.^8^; Da Silva et al.^9^). Similarly, the probiotic LBA, LAA and synbiotic LAA and LPAA samples had higher acidity values compared to the C samples with the highest value for the LPBA sample.

Tiepo et al.^18^ observed that cyanobacterial *Spirulina platensis* increased acidity and lowered pH of the ice cream samples confirming our results on the effect of the alginate-encapsulated PE addition^18^. Additionally, Sagdic et al.^11^. found that *L. casei* lowered pH and raised acidity of the ice cream samples during fermentation, confirming our results for the probiotic LBA sample. Further, the fermented ice cream's ability to retain flavour was adversely affected by the pH levels of the ice cream samples^11^. It is stated that by increasing the number of microalgae and probiotics, the amount of acid production increased, which was due to the high growth of probiotics in high concentrations of microalgae. Atik et al.^33^ fortified the vegan kefir with *Spirulina platensis and* showed that increasing the concentration of *Spirulina platensis* decreased the pH values of kefir samples^33^. In the study by Agustini et al.^34^ in evaluation of physicochemical, microbiological and sensory characteristics of yogurts enriched with *Spirulina platensis*, a decrease in pH concentration was also observed with an increase in the number of *Spirulina platensis* bacteria^34^. The same results were also seen in the study by Mocanu et al.^35^.

Ice cream is a semi-solid food that is made by freezing milk, fat, and sugar mixture with or without food additives. Commercial ice creams are deficient in protein and have antioxidant properties, are easy to melt, and are high in fat. Therefore, it is necessary to improve the quality of ice cream. Therefore, by adding nutrients with high nutritional value, such as microalgae, the nutrients of ice cream a can be provided. Since ice cream may be a major part of the diet, especially for children; the use of eatable algae paves the way to produce food rich in essential nutrients and increase their quality^36,37^. The high protein content of microalgae is one of the main reasons for considering them as the main source of protein. In our study, Protein content (%), did not differ significantly between samples (P > 0.05), confirming the findings of Da Silva et al.^9^ who found that probiotic *Bifidobacterium animalis* subsp. lactis BLC1 had no impact on protein level of the ice cream sample and contradicting those of Pandiyan et al.^13^ who found that adding *L. acidophilus* and *S. boulardii* to ice cream samples increased their protein content. Compared to our result, Shafaei Bajestani et al.^38^ showed that ice cream enriched with *Spirulina* sp. had higher protein content^38^. Morover in a study by Verga et al.^39^, the benefits of *Spirulina* biomass-enriched milk were reported. The results showed that the addition of *Spirulina* biomass increased the content of amino acids, vitamins, and fatty acids. This may be because the presence and abundance of bioactive substances in the biomass of *Spirulina* are of great nutritional importance for the production of functional dairy foods^39^. Akalin et al.^40^ in the study of the effect of *Spirulina* biomass on the microbiological activity of traditional yogurts and probiotics during refrigerated storage showed that the protein content of algae-containing samples increased significantly compared to the control sample^40^.

Microalgae are known for their high fat content, comparable to vegetable oils. While some species can accumulate up to 85% fat under specific conditions, the typical range falls between 20 and 40% of their dry weight. These fats are primarily triglycerides composed of fatty acids with chain lengths between 14 and 22 carbons. Notably, *Spirulina*, a commonly studied microalgae, boasts 5–7% lipids rich in essential fatty acids like linoleic acid and gamma-linolenic acid^41^. *Nostoc* sp. is cholesterol-free and rich in unsaturated fatty acids, which makes it suitable for treating and preventing atherosclerosis, obesity, and high blood pressure. Due to the direct effects of gamma-linolenic acid on the immune system and the treatment of many diseases, therefore, there has always been great interest in producing high concentrations of gamma-linolenic acid^42^. In the study of Bosnia et al.^43^ on the effect of concentrations of 0.25, 0.5, and 1 g/kg *S. platensis* 500 powder on traditional Greek cheese, it was shown that increasing the concentration of *Spirulina*, the amount of fat and moisture increase and decrease, respectively^43^, however, the Augustini results in 2016 showed that the application of *Spirulina platensis* powder in concentrations of 0%, 1% and 1.5% on ice cream and soft cheese did not change the fat content but reduced the moisture content^15^. The increased fat content of *Spirulina*-enriched cheese was also observed in studies by Beheshtipour et al.^43^. Tiepo et al.^18^ found that in comparison with control samples, adding 1% pure or microencapsulated *S. platensis* to ice cream increased its protein and fat content. This was expected, given the high protein and lipid content of *S. platensis* biomass. Similarly, Agustini et al.^15^ found that the addition of this cyanobacterial strain resulted in a significant effect for protein, total solid, fat and total sugar, overrun, melting point and sensory properties of ice cream samples. De Oliveira et al.^17^ found that a *Spirulina* sp. strain microencapsulated with maltodextrin and soy lecithin increased the protein content, DPPH and phenolic compounds and reduced total lipids of the chocolate milk samples.

As the consumers currently desire low-fat products, probiotic inoculation may be helpful when making low-fat ice cream. Similarly, Pandiyan et al.^13^ found that adding *L. acidophilus* and *S. boulardii* to ice cream samples lowered their fat content. However, in our study, adding encapsulated PE and bacteria had no significant deference in the fat content (%) (P > 0.05) of the ice cream samples both before and after aging.

The results of ash in our study showed a gradual increase. Compared to our study, the amount of cheese ash in the study of Beheshtipour et al.^43^, as well as Shafaei Bajestani et al.^38^ research, had increased between 10 and 11%. This increase in ash is due to the addition of PE of this microalgae due to the high percentage of micoalgal ash content. Moreover, the overrun, also known as the rise in ice cream volume, brought on by adding air, was crucial to the ice cream's quality. In our study, overrun (%), did not differ significantly between samples (P > 0.05). On the contrary, Agustini et al.^15^ found that *S. Platensis*-enriched samples had higher overrun values compared to control samples. Similarly, Da Silva et al.^9^ found that the probiotic ice cream had no change in overrun compared to C samples. The addition of extra air may have increased the mixture's volume. According to Malik et al.^16^, the ice cream prepared with the *Spirulina* biomass exhibited an increase in the overrun behaviour confirming the findings of Agustini et al.^15^.

The presence of *L. rhamnosus*, however, did not seem to have any significant impact on the overrun levels of the ice cream samples according to Alamprese et al.^8^. It is suggested that Ice cream with higher overrun would have fewer ice crystals and increases the volume of air increases after addition of solid-lipid microparticles in the ice cream. Further, it is reported that fat and protein content relatively influence ice cream overrun and contribute air interfaces stabilization^44^.

The melting characteristics of ice cream are crucial to its long-term stability and how consumers experience it in terms of texture and flavour release^4^. Melting rate is a useful indicator of structural development and resistance to collapse in ice cream as a time against average dripped volume^45^. Melting point property is attributed to water holding capacity, which refers to the interaction between protein, product, and water that results in some water remaining with the product. In a study by Priyanka Malik et al.^16^, the addition of *Spirulina* to ice cream increased the melting resistance. This may be due to the protein and fat in ice cream fortified with *Spirulina* biomass^46^. Rasouli et al.^47^ in another study optimized the formulation of traditional Iranian ice cream containing *Spirulina* microalgae^47^.

The results of their study showed that the enrichment of ice cream with *Spirulina* increases viscosity and melting resistance and reduces ice cream aeration. Based on the results of sensory evaluation, with increasing the number of *Spirulina* in the ice cream formulation, consumers' acceptance of the taste, color, texture, and overall acceptance of the product decreased. Finally, their conclusion showed that by enriching ice cream with *Spirulina* microalgae, a product with desirable physical and organoleptic properties can be achieved^47^.

It must be emphasized that in our study, the probiotic LBA and LAA and synbiotic LPBA and LPAA samples had no significant change in the melting behaviour confirming the findings of Da Silva et al.^9^. Total solid contents, ice crystal size, quantity and size of fat globules, stabilizers, emulsifiers, and storage are some factors that impact melting time. For instance, Augustini et al.^15^ found the increased melting durations for the prebiotic ice cream with *S. platensis* contradicting our findings. The greater fat content of the enriched samples may have hampered heat transfer and prolonged melting periods.

In our study, the probiotic LBA and synbiotic LPBA and LPAA samples had slightly higher viscosity values compared to the C and probiotic LAA samples. This finding shows the slight positive impact of the addition of the encapsulated PE to the synbiotic ice cream samples. In addition, there is no significant variation in the ash content of all samples (Table 2) confirming the findings of Da Silva et al.^9^. However, the moisture contents decreased across all samples (Table 4). As Pushpadass et al.^48^ showed, the moisture content of the ice cream products affects a range of physicochemical properties of the products^48^.

Regarding the overall solid content of ice cream products, Sarwar et al.^14^ found that *S. boulardii* samples had lowered overall solid content^14^. In our study, there was no significant change in the probiotic ice cream samples compared to the C samples at all freezing periods confirming Sarwar et al.^14^. However, the synbiotic LPBA and LPAA samples had higher total solid content compared to the C and probiotic LBA and LAA samples in all the freezing periods listed in Table 3. These findings show the positive slight impact of the addition of the encapsulated PE on the total solid content of the synbiotic ice cream samples.

The data presented here indicate that inoculating ice cream samples with the encapsulated PE might alter their physicochemical properties, mostly favourably. However, in comparison to the control sample, the detected modifications were not statistically significant.

*Caenorhabditis elegans* (*C. elegans*) is a favored model organism for environmental and developmental toxicology research due to several advantages. These include its abundance, ease of culture, and ability to thrive in both soil and aquatic environments. As a result, *C. elegans* provides valuable insights into the potential environmental impacts of pollutants. In this study, we employed *C. elegans* to assess the potential toxicity of PE. The results demonstrated no significant impact on *C. elegans* survival at any tested concentration (100% to 96.1% survival). This finding contrasts with the observations of Singh et al.^49^, who reported that phycocyanin (PC) extracted from *Leptolyngbya* sp. enhanced the activity of aged worms. These contrasting results highlight the need for further research to explore the potential benefits and risks associated with different types of phycobiliproteins.

Compounds containing antioxidant properties may not respond equally to different sources of free radicals. Therefore, the use of multiple assays to measure antioxidant capacity is necessary for appropriate assessments. It was shown that the reducing power of the FRAP method is dose-dependent and with increasing the concentration of phycocyanin, the antioxidant activity increases^50^. Similar results were observed in the study of the antioxidant and nutritional potential of cookies enriched with Biomass *Spirulina platensis* at concentrations of 2% and 5% by the FRAP method by Egea et al.^51^. Research conducted by Gabr et al.^52^ studies the antioxidant activity of phycocyanin extracted from *S. platensis* at concentrations of 5,10,15,20,25,30 µg/ml by the DPPH method showed that As the concentration of phycocyanin increases, its antioxidant activity increases. Shalaby et al.^53^ attributed the antioxidant activity of *Spirulina* extracts by DPPH to the presence of phenols with high levels of biologically active phytochemicals (sterols, flavonoids, reducing sugars, tannins, and anthraquinones)^53^. In addition, in a study on the evaluation of antioxidant activity by nitric oxide trapping method of *S. platensis* ethanolic extract in vitro, it was shown that its antioxidant activity is dose-dependent, and with increasing the concentration of the extract, the antioxidant activity increases^54^; Similar results were obtained in the study of extraction and purification of phycocyanin from the dry powder of *Spirulina platensis* and evaluation of its antioxidant, anticoagulant activity and prevention of DNA damage^55^.

The antioxidant properties of yogurt and cheese enriched with *Spirulina* were investigated by Barkala et al.^56^. In their study, after adding *Spirulina* to yogurt in different concentrations, they studied its effect on the fermentation process, texture, and nutritional and sensory properties of yogurt. The data showed that the addition of *Spirulina* biomass accelerates fermentation and preserves our tissue properties and sensory acceptance^56^.

According to the EC_50_ value, a sample's intensity should result in a 50% drop in the original DPPH concentration. While low EC_50_ values demonstrate considerable antioxidant activity, high EC_50_ values suggest poor antioxidant activity. Our findings suggested that antioxidant activity was considerably reduced to eight weeks of freezing at − 20 °C in all samples (P < 0.05) (Table 5). Similarly, Sagdic et al.^26^ found that after being frozen for 60 days, ice cream lost its ability to scavenge DPPH radicals confirming our findings. Further, the synbiotic LPBA samples had the highest antioxidant activity compared to C, LPA, LAA, and LPAA samples across all the freezing periods. In other words, the addition of the encapsulated PE resulted in the increasing antioxidant activity for the synbiotic ice cream samples both before and after aging. This finding justifies the use of the encapsulated PE in the ice cream products making them healthier for the consumers.

The most important finding in this respect is that both probiotic (LBA and LAA) and synbiotic (LPBA and LPAA) samples had more bacterial counts for all freezing periods from day one to two weeks. Secondly, the LAA and LPAA samples had less bacterial counts in relation to the respective LBA and LPBA samples. Thirdly, all samples had less bacterial counts after the six and eight weeks of freezing.

Fourthly, the use of the encapsulated PE resulted in the increased bacterial counts in the synbiotic LPBA and LPAA samples compared to probiotic samples, indicating the positive impact of the PE addition to the ice cream products. Similarly, the inulin and fructo-oligosaccharide prebiotics increased the viability of *L. acidophilus* and *S. boulardii* in ice cream samples^13,14^, confirming our findings in this respect.

The third finding implies that that viable bacterial populations could decrease by at least 1 log unit during eight weeks of freezing. Furthermore, storing at a low temperature (− 18 °C) might have contributed to the declining viable bacteria numbers during freezing.

It has also been recently documented that the viable counts of *L. acidophilus*, *S. boulardii* and *L. rhamnosus* decreased in frozen storage^13,14,57^ confirming our third finding. Bacterial numbers might have decreased because the mixture was stirred and had access to oxygen^10^. However, Abghari et al.^7^ showed that the viable count of *L. rhamnosus* did not change noticeably throughout the storage period of 12 weeks^7^.

As Mohammadi et al.^5^ suggests one way to preserve the bacterial populations after the eight weeks of freezing is the encapsulation of the bacteria as it was done in Li et al.^58^ following the case of the encapsulation of PE with alginate in this study and encapsulation of *Spirulina* biomass^17,18^. They encapsulated *L. rhamnosus* using pectin, glucose, and calcium chloride. They found that encapsulation increased tolerance of bacteria in the acid condition, protected them from protease digestion, and improved shelf time when stored at the ambient condition.

Similarly, the probiotic and synbiotic ice cream samples with *L. casei* and *L. rhamnosus* and inulin had bacterial populations above the minimal numbers required for a probiotic sample after eight weeks of freezing at − 20 °C^6^, confirming our findings. As predicted, in our sample the probiotic content of ice cream products decreased throughout storage, but more crucially, none had probiotics below 6.18 log CFU mL^−1^. The freezing process, which damaged cells and ultimately led to cell death, possibly caused the decline of probiotics after eight weeks of freezing^5,11^.

Color in food has an important impact on consumers since it is one of the first characteristics we perceive from a product. Additionally, colored food is attractive, and color allows for better identification and selection among similar products. Coloration helps to relate water with food; for example, yogurt, animal or vegetal milk (e.g., soybean, coconut, or almonds) with fruits such as strawberries (reddish), blue berries (purple), and melon (green). Then, consumers would consider ingesting these types of food as beneficial to their well-being even though they may not have fruits^59^. Cyanobacterial PBPs are natural pigments used as colorants in some food products; for example, aqueous extracts of non-purified blue PC from *Spirulina* have been added in ice creams, yogurts, isotonic beverages, confectionery, and jellies^60^. Particularly, bright blue PC has been selected over others less-bright natural colorants such as gardenia blue and indigo in confectionery production^61–63^. Red PE has been mostly used as a fluorescent probe in biomedical studies, rather than in the food industry. The addition of microalgal biomass and their derivatives such as PE to ice cream, can change the sensory qualities such as colour, aroma, flavour, taste, and texture of the product. Sensory characteristics play an important role in the overall acceptance of food products by consumers. Therefore, consumers should avoid functional foods if the combined ingredients produce unpleasant tastes. The addition of microalgae creates an unpleasant odor similar to fish, which is due to its mineral content^15^. Researchers have studied methods to reduce the odor associated with *Spirulina* using activated charcoal adsorption, heating, enzymatic hydrolysis of lysozyme, the inclusion of beta-cyclodextrin, fermentation and solvent extraction, and fermentation and extraction of ethanol as the most effective method to reduce the odor associated with *Spirulina* has been also proved^64^.

According to Durmaz et al.^4^, adding *P. cruentum* enhanced the ice cream's aesthetics because of the pink pigments found in the microalgae. Nevertheless, the panellists felt that high levels of microalgae might harm sensory qualities. Since the panellists agreed that the bitter taste was stronger, adding more microalgae had a negative impact on the ice cream flavour. In comparison, the probiotic LBA sample was the one with the best flavour confirming the findings of Di Criscio et al.^6^. Further, compared to the C sample, the synbiotic LPBA and LPAA samples had poorer ratings for aroma, flavour, colour, texture, and overall acceptability attributes (P ≤ 0.05) confirming the findings of Di Criscio et al.^6^. However, there was a positive impact of the microencapsulation of *S. platensis* with Arabic gum or maltodextrin on the acceptability ratings^18^.

It can be stated that changes in texture is related to the fatty acid composition of the product, The products enriched with PE may have lowered the saturated fatty acids, such as C14:0 and increased the unsaturated fatty acids, such as C18:1^65^. Overall, the sensory characteristics largely rely on the raw materials and production techniques used. Further investigation is required to evaluate the influence of diverse components, including VOCs, AAs, FAs, and peptides, on each sensory attribute. In another study by Lambiase et al.^66^, the sensory evaluation of cheese with *Spirulina* sp. Were performed. The results showed that *Spirulina*-mozzarella cheeses were perceived with a higher butter odor, a higher whey flavor, and a sweeter and bitterer taste than the samples from the control group. Furthermore, the panelists perceived higher oiliness and moisture intensities in the samples with *Spirulina* with a greater milk release when cutting and a tendentially higher tenderness than in the mozzarella cheeses from the control group, which, on the contrary, were perceived as being more grainy, cohesive, screechy, and having a greater consistency when cutting than the *Spirulina*-mozzarella cheeses.

Volatile organic compounds (VOCs) are chemical substances in nature and plant cultivation. Microalgae, a is one of the most VOC-rich substances. There are some different types of cyanobacteria VOCs components that have been discovered, and some of them are responsible for the authentic aroma such as β-ionone, heptadecane and dihydrvactinodiolide^67^. The environmental and taxonomical factors determine the composition of chemical content and VOC in microalgae biomass^68^. The environmental factor of cultivation such as, temperature, salinity, illumination, pH, nutrient and cultivation methods^69^. Composition of VOC in substances detection by Gas Chromatography (GC) separation techniques. Gas Chromatography (GC) is a technique for separating chemicals found in a sample. The retention time (RT) is the time it takes for a certain compound to transit the length of the column and is the defining property^70^.

When microalgal biomass is added to food products, its odor may influence the sensory properties of the final products. However, increasing the addition of microalgae in food products can improve the contain of bioactive compounds and antioxidant capacity but decrease the panelist acceptance^51,71^. The consumer acceptance and purchase intention of a food product will be primarily affected by odor and flavor. For example, The fortified dark chocolate with *Spirulina* has consumer preferences with lowest value (55.8%)^72^. The purchase intention consumers of the cookies fortified by *Spirulina* have lower values than formulation without auditioning *Spirulina*. The fishy odor of these volatile components reduces consumer acceptance and inhibits the development of edible algae^73,74^.

The inoculation stage, inoculation technique, co-inoculation of probiotics, and the range of volatile ingredients present in the synbiotic ice cream samples all influenced the volatile profile of ice cream products. Furthermore, given that the aging process is crucial for the sensory evaluation of ice cream products, these results implied that *L. casei* infection occurred before synbiotic ice cream production. Major aromatic components that significantly contribute to the aroma character of functional products include aldehydes, ketones, and esters^14^.

In this study, the VC profiles of C and synbiotic LPBA samples of ice cream were evaluated before the assessment of aging after one week of storage. The Table 7 shows that the major VCs were acetone and ethanol with over 23 and 15% of the C sample and the content of these VCs fell significantly in the PLBA sample compared to the C sample.

## Conclusion

This is the first study, testing the usage of PE as a natural pigment extracted from *Nostoc* sp. together with a probiotic *Lactobacillus* sp. taking into account the aging process during probiotic ice cream production. In this perspective, PE from a *Nostoc* cyanobacterial strain encapsulated with alginate was introduced as potential prebiotics to produce synbiotic ice cream products with *L. casei*. The addition of the encapsulated PE affected, mostly favourable, the physicochemical properties, antioxidant activity, probiotic survival, VC contents, and sensory acceptability of the synbiotic ice cream samples before and after aging at the freezing periods from one day to eight weeks. The results of this study opens the ways for further research on using the PEs and other PBPs from other cyanobacterial and other algal strains encapsulated with alginate and other encapsulating materials to produce synbiotic ice cream and other dairy products using *Lactobacilli* and other microbial strains. As discussed in this paper, the research on the encapsulation of the bacteria could be integrated with such studies to protect the integrity of the bacteria in the harsh processing conditions of ice cream production and storage.

### Supplementary Information

Supplementary Information.

## Data Availability

The datasets used and/or analysed included in this published article [As supplemtray file, Supp. S1].

## Abbreviations

PE: Phycoerythrin
PBPs: Phycobiliproteins
B-PE: B-phycoerythrin
R-PE: R-phycoerythrin
C-PE: C-phycoerythrin
GIT: Gastrointestinal tract
IDF: International dairy federation
PE: Phycoerythrin
PC: Phycocyanin
APC: Allophycocyanin
PBPs: Biliproteins
DSC: *Differential scanning calorimetry*
GAE: Gallic acid equivalents
DPPH: 1,1-Diphenyl-2-picrylhydrazyl radical
GC-MS: Gas chromatography-mass spectrometry
NIST: National Institute Standard, and Technology
WHO: World Health Organization

## References

[1] Bhowmik D, Dubey J, Mehra S (2009). Probiotic efficiency of Spirulina platensis-stimulating growth of lactic acid bacteria. World J. Dairy Food Sci..

[2] García AB, Longo E, Murillo MC, Bermejo R (2021). Using a B-phycoerythrin extract as a natural colorant: Application in milk-based products. Molecules..

[3] Chen H, Qi H, Xiong P (2022). Phycobiliproteins: A family of algae-derived biliproteins: Productions, characterization and pharmaceutical potentials. Mar. Drugs..

[4] Durmaz Y, Kilicli M, Toker OS, Konar N, Palabiyik I, Tamtürk F (2020). Using spray-dried microalgae in ice cream formulation as a natural colorant: Effect on physicochemical and functional properties. Algal Res..

[5] Mohammadi R, Mortazavian AM, Khosrokhavar R, da Cruz AG (2011). Probiotic ice cream: Viability of probiotic bacteria and sensory properties. Ann. Microbiol..

[6] Di Criscio T, Fratianni A, Mignogna R (2010). Production of functional probiotic, prebiotic, and synbiotic ice creams. J. Dairy Sci..

[7] Abghari A, Sheikh-Zeinoddin M, Soleimanian-Zad S (2011). Nonfermented ice cream as a carrier for *Lactobacillus acidophilus* and *Lactobacillus rhamnosus*. Int. J. Food Sci. Technol..

[8] Alamprese C, Foschino R, Rossi M, Pompei C, Corti S (2005). Effects of *Lactobacillus rhamnosus* GG addition in ice cream. Int. J. Dairy Technol..

[9] Da Silva PDL, de Fátima BM, dos Santos KMO, Correia RTP (2015). Potentially probiotic ice cream from goat's milk: Characterization and cell viability during processing, storage and simulated gastrointestinal conditions. LWT-Food Sci. Technol..

[10] Hagen M, Narvhus J (1999). Production of Ice Cream Containing Probiotic Bacteria.

[11] Homayouni A, Azizi A, Javadi M, Mahdipour S, Ejtahed H (2012). Factors influencing probiotic survival in ice cream: A review. Int. J. Dairy Technol..

[12] Gupta S, Gupta C, Garg A, Prakash D (2017). Prebiotic efficiency of blue green algae on probiotics microorganisms. J. Microbiol. Exp..

[13] Pandiyan C, Annal Villi R, Kumaresan G, Murugan B, Gopalakrishnamurthy T (2012). Development of synbiotic ice cream incorporating *Lactobacillus acidophilus* and *Saccharomyces boulardii*. Int. Food Res. J..

[14] Sarwar A, Aziz T, Al-Dalali S (2021). Characterization of synbiotic ice cream made with probiotic yeast *Saccharomyces boulardii* CNCM I-745 in combination with inulin. LWT..

[15] Winarni Agustini T, Farid Ma’ruf W, Widayat W, Suzery M, Hadiyanto H, Benjakul S (2016). Application of spirulina platensis on ice cream and soft cheese with respect to their nutritional and sensory perspectives. J. Teknol..

[16] Malik P, Kempanna C, Paul A (2013). Quality characteristics of ice cream enriched with Spirulina powder. Int. J. Food Nutr. Sci..

[17] De Oliveira TTB, dos Reis IM, de Souza MB (2021). Microencapsulation of *Spirulina* sp. LEB-18 and its incorporation in chocolate milk: Properties and functional potential. LWT..

[18] Tiepo CBV, Gottardo FM, Mortari LM, Bertol CD, Reinehr CO, Colla LM (2021). Addition of Spirulina platensis in handmade ice cream: Phisicochemical and sensory effects/Adição de Spirulina platensis em sorvete caseiro: Efeitos físico-químicos e sensoriais. Braz. J. Dev..

[19] Ganesan AR, Shanmugam M (2020). Isolation of phycoerythrin from *Kappaphycus alvarezii*: A potential natural colourant in ice cream. J. Appl. Phycol..

[20] Ramu Ganesan A, Kannan M, Karthick Rajan D (2023). Phycoerythrin: A pink pigment from red sources (rhodophyta) for a greener biorefining approach to food applications. Crit. Rev. Food Sci. Nutr..

[21] Afreen S, Fatma T (2018). Extraction, purification and characterization of phycoerythrin from Michrochaete and its biological activities. Biocatal. Agric. Biotechnol..

[22] Nowruzi, B., Fahimi, H. & Lorenzi, A. S. Recovery of pure C-phycoerythrin from a limestone drought tolerant cyanobacterium Nostoc sp. and evaluation of its biological activity. *Anal. Biol*. (2020).

[23] Galetović A, Seura F, Gallardo V (2020). Use of phycobiliproteins from atacama cyanobacteria as food colorants in a dairy beverage prototype. Foods..

[24] Horwitz, W. & Latimer, G. W. *Official Methods of Analysis of AOAC International*, vol. 1 (AOAC International, 2000).

[25] Molina MI, Petruccelli S, Añón MC (2004). Effect of pH and ionic strength modifications on thermal denaturation of the 11S globulin of sunflower (*Helianthus annuus*). J. Agric. Food Chem..

[26] Sagdic O, Ozturk I, Cankurt H, Tornuk F (2012). Interaction between some phenolic compounds and probiotic bacterium in functional ice cream production. Food Bioprocess Technol..

[27] Čanadanović-Brunet JM, Ćetković GS, Djilas SM (2009). Radical scavenging and antimicrobial activity of horsetail (*Equisetum arvense* L.) extracts. Int. J. Food Sci. Technol..

[28] Kumar T, Jain V (2015). Appraisal of total phenol, flavonoid contents, and antioxidant potential of folkloric *Lannea coromandelica* using in vitro and in vivo assays. Scientifica..

[29] Lawless HT, Heymann H (2010). Sensory Evaluation of Food: Principles and Practices.

[30] Zheng J, Wittouck S, Salvetti E (2020). A taxonomic note on the genus *Lactobacillus*: Description of 23 novel genera, emended description of the genus *Lactobacillus* Beijerinck 1901, and union of *Lactobacillaceae* and *Leuconostocaceae*. Int. J. Syst. Evol. Microbiol..

[31] Tan HT, Yusoff FM, Khaw YS (2022). A review on a hidden gem: Phycoerythrin from blue-green algae. Mar. Drugs..

[32] Nowruzi B, Anvar A, Ahari H (2020). Extraction, purification and evaluation of antimicrobial and antioxidant properties of phycoerythrin from terrestrial cyanobacterium *Nostoc* sp. FA1. J. Microb. World..

[33] Atik DS, Gürbüz B, Bölük E, Palabıyık İ (2021). Development of vegan kefir fortified with *Spirulina platensis*. Food Biosci..

[34] Agustini T, Soetrisnanto D, Ma’ruf W (2017). Study on chemical, physical, microbiological and sensory of yoghurt enriched by *Spirulina platensis*. Int. Food Res. J..

[35] Mocanu G, Botez E, Nistor OV, Andronoiu D, Vlăsceanu G (2013). Influence of Spirulina platensis biomass over some starter culture of lactic bacteria. J. Agroaliment. Process. Technol..

[36] Anvar, S. A. A., Nowruzi, B. & Afshari, G. *A Review of the Application of Nanoparticles Biosynthesized by Microalgae and Cyanobacteria in Medical and Veterinary Sciences*. (2023).

[37] Pourbaba, H., Anvar, A. A., Pourahmad, R. & Ahari, H. Changes in acidity parameters and probiotic survival of the kefir using *Lactobacillus acidophilus* and *Lactobacillus paracasei* complementary probiotics during cold preservation. (2022).

[38] Shafaei Bejestani M, Anvar AA, Nowruzi B, Golestan L (2023). Production of cheese and ice cream enriched with biomass and supernatant of Spirulina platensis with emphasis on organoleptic and nutritional properties. Iran. J. Vet. Med..

[39] Varga L, Szigeti J, Kovács R, Földes T, Buti S (2002). Influence of a Spirulina platensis biomass on the microflora of fermented ABT milks during storage (R1). J. Dairy Sci..

[40] Akalin A, Unal G, Dalay M (2009). Influence of Spirulina platensis biomass on microbiological viability in traditional and probiotic yogurts during refrigerated storage. Italian J. Food Sci..

[41] Ötleş S, Pire R (2001). Fatty acid composition of Chlorella and *Spirulina microalgae* species. J. AOAC Int..

[42] Sajilata M, Singhal RS, Kamat MY (2008). Supercritical CO_2_ extraction of γ-linolenic acid (GLA) from Spirulina platensis ARM 740 using response surface methodology. J. Food Eng..

[43] Bosnea, L. *et al*. Incorporation of Spirulina platensis on traditional greek soft cheese with respect to its nutritional and sensory perspectives. *Proceedings* (2020).

[44] Flores A, Goff H (1999). Ice crystal size distributions in dynamically frozen model solutions and ice cream as affected by stabilizers. J. Dairy Sci..

[45] Chen W, Liang G, Li X (2019). Effects of soy proteins and hydrolysates on fat globule coalescence and meltdown properties of ice cream. Food Hydrocoll..

[46] Sofjan RP, Hartel RW (2004). Effects of overrun on structural and physical characteristics of ice cream. Int. Dairy.

[47] Rasoulu, F., Berenji, S. & Shahab, L. A. *Optimization of Traditional Iranian Ice Cream Formulation Enriched with Spirulina Using Response Surface Methodology*. (2017).

[48] Pushpadass HA, Mitra H, Franklin MEE, Ghoroi C, Ambrose RPK, Battula SN (2021). Physicochemical, thermal, and flow properties of ice cream powder as influenced by moisture content. J. Food Process. Preserv..

[49] Singh NK, Sonani RR, Awasthi A (2016). Phycocyanin moderates aging and proteotoxicity in *Caenorhabditis elegans*. J. Appl. Phycol..

[50] Punampalam R, Khoo KS, Sit NW (2018). Evaluation of antioxidant properties of phycobiliproteins and phenolic compounds extracted from *Bangia atropurpurea*. Malay. J. Fundam. Appl. Sci..

[51] Egea B, Campos A, De Carvalho-Eliane JCM, Danesi DG (2014). Antioxidant and nutritional potential of cookies enriched with Spirulina platensis and sources of fibre. J. Food Nutr. Res..

[52] Gabr GA, El-Sayed SM, Hikal MS (2020). Antioxidant activities of phycocyanin: A bioactive compound from *Spirulina platensis*. J. Pharm. Res. Int..

[53] Shalaby, E. A. & Shanab, S. M. *Comparison of DPPH and ABTS Assays for Determining Antioxidant Potential of Water and Methanol Extracts of Spirulina platensis*. (2013).

[54] Anbarasan V, Kumar VK, Kumar PS, Venkatachalam T (2011). In vitro evaluation of antioxidant activity of blue green algae *Spirulina platensis*. Int. J. Pharm. Sci. Res..

[55] Kamble SP, Gaikar RB, Padalia RB, Shinde KD (2013). Extraction and purification of C-phycocyanin from dry Spirulina powder and evaluating its antioxidant, anticoagulation and prevention of DNA damage activity. J. Appl. Pharm. Sci..

[56] Barkallah M, Dammak M, Louati I (2017). Effect of *Spirulina platensis* fortification on physicochemical, textural, antioxidant and sensory properties of yogurt during fermentation and storage. LWT..

[57] Heenan C, Adams M, Hosken R, Fleet G (2004). Survival and sensory acceptability of probiotic microorganisms in a nonfermented frozen vegetarian dessert. LWT-Food Sci. Technol..

[58] Li R, Zhang Y, Polk DB, Tomasula PM, Yan F, Liu L (2016). Preserving viability of *Lactobacillus rhamnosus* GG in vitro and in vivo by a new encapsulation system. J. Control. Release..

[59] Griffiths JC (2005). Coloring foods & beverages. Food Technol..

[60] Food U, Administration D. *Summary of Color Additives for Use in the United States in Foods, Drugs, Cosmetics, and Medical Devices*. (US Department of Health and Human Services, 2017).

[61] Jespersen L, Strømdahl LD, Olsen K, Skibsted LH (2005). Heat and light stability of three natural blue colorants for use in confectionery and beverages. Euro. Food Res. Technol..

[62] Pandey V, Pandey A, Sharma V (2013). Biotechnological applications of cyanobacterial phycobiliproteins. Int. J. Curr. Microbiol. Appl. Sci..

[63] Sharma NK, Rai AK, Stal LJ (2013). Cyanobacteria: An Economic Perspective.

[64] Bao J, Zhang X, Zheng J-H, Ren D-F, Lu J (2018). Mixed fermentation of *Spirulina platensis* with *Lactobacillus plantarum* and *Bacillus subtilis* by random-centroid optimization. Food Chem..

[65] Uzun P, Masucci F, Serrapica F (2018). The inclusion of fresh forage in the lactating buffalo diet affects fatty acid and sensory profile of mozzarella cheese. J. Dairy Sci..

[66] Lambiase C, Braghieri A, Barone CMA (2023). Use of cyanobacterium spirulina (*Arthrospira platensis*) in buffalo feeding: Effect on mozzarella cheese quality. Foods..

[67] Aguero J, Lora J, Estrada K (2003). Volatile components of a commercial sample of the blue-green algae Spirulina platensis. J. Essen. Oil Res..

[68] Prasetiyo, H. *et al*. Off-odour identification from volatile organic compounds (VOCs) of Spirulina. in *BIO Web of Conferences* (2024).

[69] Afriani S, Uju U, Setyaningsih I (2018). Chemical composition of spirulina platensis which cultivated in photobioreactors with different photoperiodes. Jurnal Pengolahan Hasil Perikanan Indon..

[70] Liu L, Liu X, Jia J (2022). Economic analysis of volatile characteristics of *Haematococcus pluvialis* and the effect of roasting temperature on the profile of volatiles and chemical components. Algal Res..

[71] Boyanova, P. *et al*. Effect of *Spirulina platensis* on the quality and antioxidants characteristics of ice cream. in *BIO Web of Conferences* (2022).

[72] Asti, G. K. & Ekantari, N. Consumer preferences for dark chocolate products fortified with *Spirulina platensis* using analytical hierarchy process method. in *E3S Web Of Conferences* (2020).

[73] Hamdan, A. B., Riaty, C., Fitriya, W. & Ekantari, N. Effects of nanoencapsulated carotenoid of *Spirulina platensis* on the sensory profiles of dark and milk chocolate. in *E3S Web Of Conferences* (2020).

[74] Wang J, Zhang M, Fang Z (2019). Recent development in efficient processing technology for edible algae: A review. Trends Food Sci. Technol..

